# Photodynamic therapy of melanoma by blue-light photoactivation of flavin mononucleotide

**DOI:** 10.1038/s41598-019-46115-w

**Published:** 2019-07-04

**Authors:** R. A. Akasov, N. V. Sholina, D. A. Khochenkov, A. V. Alova, P. V. Gorelkin, A. S. Erofeev, A. N. Generalova, E. V. Khaydukov

**Affiliations:** 1 I.M. Sechenov First Moscow State Medical University, 119991 Trubetskaya str. 8-2, Moscow, Russia; 2 Shemyakin - Ovchinnikov Institute of Bioorganic Chemistry Russian Academy of Sciences, 117997 Miklukho-Maklaya str. 16/10, Moscow, Russia; 3 Federal Scientific Research Center «Crystallography and Photonics» Russian Academy of Sciences, 119333 Leninskiy Prospekt 59, Moscow, Russia; 4 FSBSI “N.N. Blokhin National medical research center for oncology” of Ministry of Health of the Russian Federation, 115478 Kashirskoe Shosse 24, Moscow, Russia; 5 Lomonosov Moscow State University, 119991 Leninskiye Gory 1-3, Moscow, Russia; 6 Medical Nanotechnology LLC, Stroiteley 4-5-47, 119311 Moscow, Russia; 7 National University of Science and Technology «MISIS», Leninskiy Prospect 4, 119991 Moscow, Russia; 8 Togliatti State University, 445020 Belorusskaya str. 14, Togliatti, Russia; 9 Volgograd State University, 400062 Universitetskiy Prospect, 100, Volgograd, Russia

**Keywords:** Cancer therapy, Melanoma

## Abstract

Melanoma is one of the most aggressive and lethal form of cancer. Photodynamic therapy (PDT) is a clinically approved technique for cancer treatment, including non-melanoma skin cancer. However, the most of conventional photosensitizers are of low efficacy against melanoma due to the possible dark toxicity at high drug concentrations, melanin pigmentation, and induction of anti-oxidant defense mechanisms. In the current research we propose non-toxic flavin mononucleotide (FMN), which is a water-soluble form of riboflavin (vitamin B2) as a promising agent for photodynamic therapy of melanoma. We demonstrated selective accumulation of FMN in melanoma cells *in vivo* and *in vitro* in comparison with keratinocytes and fibroblasts. Blue light irradiation with dose 5 J/cm^2^ of melanoma cells pre-incubated with FMN led to cell death through apoptosis. Thus, the IC_50_ values of human melanoma A375, Mel IL, and Mel Z cells were in a range of FMN concentration 10–30 µM that can be achieved in tumor tissue under systemic administration. The efficiency of reactive oxygen species (ROS) generation under FMN blue light irradiation was measured in single melanoma cells by a label-free technique using an electrochemical nanoprobe in a real-time control manner. Melanoma xenograft regression in mice was observed as a result of intravenous injection of FMN followed by blue-light irradiation of tumor site. The inhibition of tumor growth was 85–90% within 50 days after PDT treatment.

## Introduction

Melanoma is one of the most aggressive and lethal form of cancer due to its metastasis capacity and acquired resistance to chemotherapy^1^. Although melanoma is a rare tumor that accounts for less than 4% of skin cancer cases, it is responsible for 80% of skin cancer deaths^2^. Melanoma occurs as a result of malignant transformation of melanocytes^3,4^ that can give rise to a diverse set of neoplasms with various biochemical cascade mutations, histopathological appearance, and clinical features^5,6^. Current anti-melanoma therapy involves tumor excision and lymph node management for early stages, while for unresectable and recurrent melanoma a number of immunotherapy (interleukin-2, pembrolizumab, nivolumab and ipilimumab) and chemotherapy (dacarbazine and temozolomide) agents are used^7^. However, existing therapeutic strategies have shown little effect against metastatic melanoma and cause serious side effects owing to nonspecific targeting of the drugs^8^. Several targeted anti-melanoma agents, which are specific to one of the melanoma sub-types, have been proposed recently, including BRAF inhibitors, MEK inhibitors, c-Kit inhibitors, and so on^9,10^. However, since personalized biochemical and genetic profiling in clinics is still challenging, there is a need in an agent with a broad specificity to various melanoma sub-types.

Photodynamic therapy (PDT) is a clinically approved, minimally invasive therapeutic procedure based on selective photosensitizer (PS) localization in neoplastic cells and vasculature with subsequent generation of reactive oxygen species (ROS) under light irradiation^11–13^. Following light excitation in the absorbance band of sensitizer, photoexcited in long-lived triplet state molecules can initiate two kinds of reactions. First type is direct transfer the energy from sensitizer to oxygen molecules dissolved in tissue and generation singlet oxygen toxic to cells. Another pathway of the induced phototoxicity to cells is direct reaction with a cell membrane or other molecule, and transfer a hydrogen atom (electron) to form radicals which interact with oxygen and produce oxygenated products^14^. Up to date, PDT was of limited efficacy against melanoma due to high dark toxicity of the most common photosensitizers, melanin pigmentation, and anti-oxidant defense mechanisms in melanoma cells. However, since localized cutaneous melanoma is a skin tumor, which is easy to irradiate, PDT is still one of the promising therapeutic procedures. Due to a control of the light exposure location and PS activation on-demand, PDT enables rather good selective anti-tumor activity and low side effects in comparison with conventional radiotherapy and chemotherapy. Furthermore, PDT stimulates anti-tumor immunity and leads to the development of an immune memory to prevent tumor recurrence^15^.

Photosensitizers like a chlorin e6^16^ or verteporfin^17,18^ have been shown as effective anti-melanoma agents in clinics, and a number of novel PSs are under laboratory investigations. However, most of the currently used PSs are organic dyes that suffer from negative side effects, poor water solubility, and low tumor targeting efficacy^19^. Moreover, most of PSs are prone to photobleaching and require the PS reinjection after irradiation step, while the number of injection-irradiation cycles is limited by the PSs systemic dark toxicity. As a result, the PDT can be insufficient for tumor regression.

Riboflavin or vitamin B2 is a cofactor for a variety of flavoprotein enzyme reactions that can be discussed as an endogenous photosensitizer^20^ that generates more singlet oxygen than exogenous PSs, such as clinically used Photofrin^21^. Due to its photochemical properties and ability to form ROS under UV- and blue-light irradiation, riboflavin (Rf) was demonstrated as an efficient antibacterial^22^ and antivirus agent^23^. Recently, Rf has been discussed as a photosensitizer for cervical cancer HeLa cells treatment *in vitro*^24^. The inhibitory effect of Rf on the growth of B16 melanoma cells was also described earlier^25^. Additionally, Rf was proposed previously as an adjuvant for cisplatin therapy of skin cancer^26^.

Riboflavin, as well as its water-soluble form flavin mononucleotide, has two absorption lines centered at 375 and 450 nm^27^. Light penetration depth at 450 nm was estimated as 1.5–2 mm^28^, which is generally not enough to achieve tumor cells since even melanoma is about 3 mm in depth for grade IV–V^29^. However, unlike conventional PSs, FMN is generally recognized as safe and practically non-toxic at least up to 10 g/kg of oral administration to rats^30^. Therefore, FMN can be re-injected several times without systemic toxic effects. Moreover, being a cofactor for a variety of flavoprotein enzyme reactions, FMN is not recognized by human ABC transporters as an exogenous drug, so FMN can overcome drug resistance often occurs in tumors.

To date, Rf has not been discussed in terms of selective anti-melanoma properties and blue-light induced ROS formation in melanoma cells. Within this context, we propose FMN as a targeting phototherapy agent for melanoma treatment. Aiming to quantify activity of photosensitizer in real-time control as a function of cell-specific antioxidant defense system, we utilized a nanoelectrode for single cell label-free ROS measurement. Finally, we administrated FMN via intravenous injection followed by PDT treatment, and melanoma xenograft regression in mice was observed.

## Results and Discussion

### Accumulation

**Figure 1:**
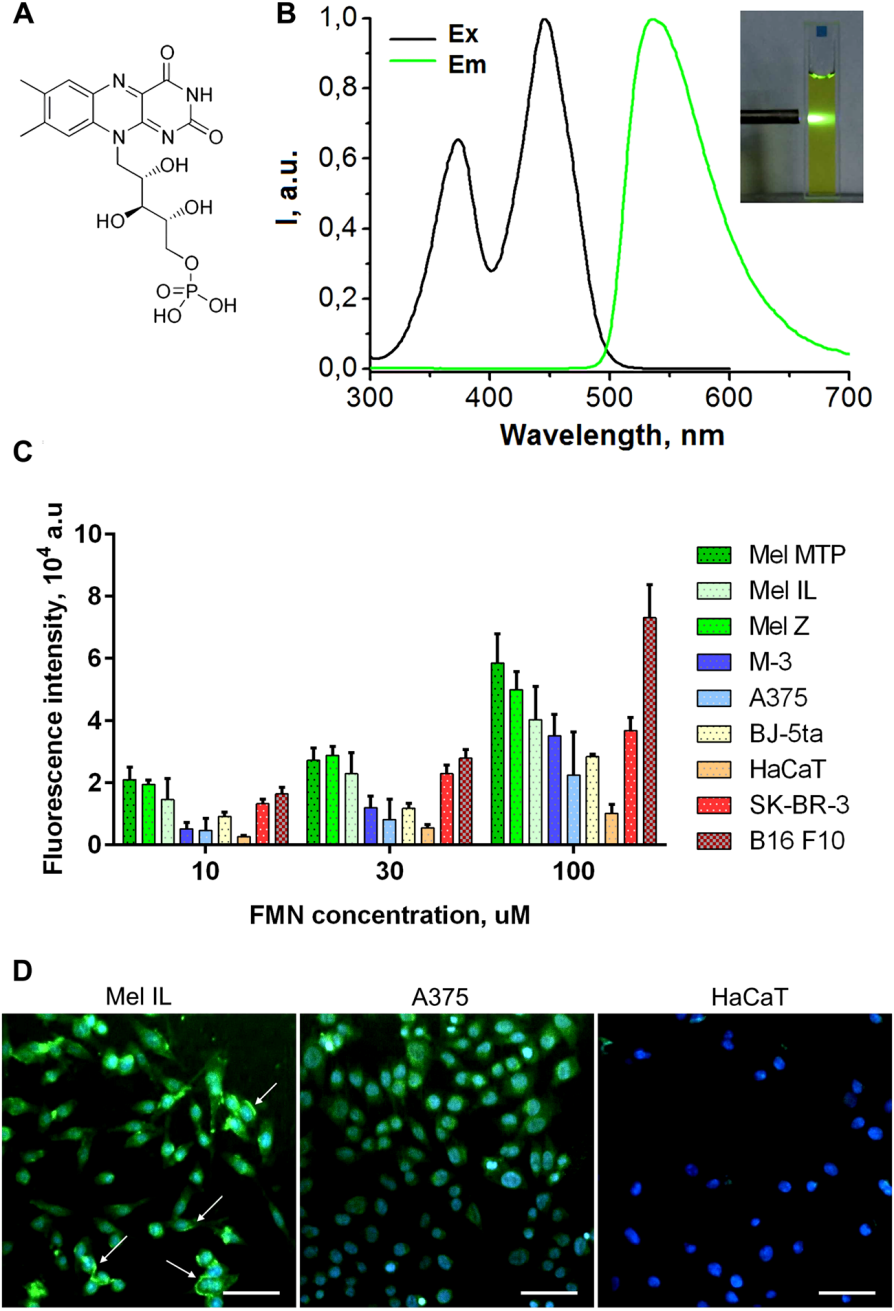
The formula (**A**) and the excitation and emission spectra of FMN in PBS (pH 7.4) (**B**); photoluminescence of FMN under 450 nm excitation are shown in insert. The accumulation of FMN in tumor and normal cells: flow cytometry data (**C**) and confocal microscopy (**D**). Flow cytometry measurements were performed at least in three independent experiments, and the data were expressed as mean ± SD. For confocal imaging, cells were incubated with 100 µM FMN solution for 30 min. Cell nucleuses are in blue (Hoechst 33258), FMN is in green. White arrows show the FMN accumulation on the cell membrane. Scale bar is 50 µm.

The accumulation of FMN was evaluated by flow cytometry using seven tumor (human melanoma Mel MTP, Mel IL, Mel Z, A375, mouse melanoma M-3, mouse melanoma B16 F10, and human breast adenocarcinoma SK-BR-3) and two normal (human keratinocytes HaCaT and human skin fibroblasts BJ-5ta) cell lines. The fluorescence was normalized to the background signal of each cell line. It was found that all melanoma cells demonstrated higher FMN accumulation compared to the human keratinocytes HaCaT (Fig. 1). Moreover, five of six melanoma cell lines, namely Mel MTP, Mel IL, Mel Z, B16 F10, and M-3, accumulated more FMN then BJ-5ta fibroblasts (FMN range was 10–100 µM), while FMN accumulation in A375 cells was of similar level. Human breast adenocarcinoma SK-BR-3 cells were proposed as a positive control since high riboflavin accumulation was demonstrated for this cell line earlier^31^. Human melanoma cells Mel MTP, Mel IL, and Mel Z accumulated more FMN then SK-BR-3 cells. All of these findings confirm the idea that FMN can be proposed for melanoma cells targeting. Additionally, confocal fluorescent images of Mel IL cells showed the FMN accumulation on the cell membrane – probably, due to the FMN interaction with cell surface receptors (Fig. 1D, Supplementary Data 1). It should be noted, target properties of riboflavin molecule were demonstrated earlier for other types of tumors including breast cancer^32^, cervical cancer^33,34^, or prostate cancer^35^ cells. Moreover, intracellular fluorescence, occurred as a result of riboflavin accumulation in membrane-bounded cytoplasmic structures bearing ATP-dependent ABCG2 transporters, has been reported earlier as a biomarker for epithelial cancer stem cells^36^. This is in a good correlation with the fact that Mel MTP and Mel IL cells contain increased quantity of cells positive for cancer stem cells markers (CD44 and CD133 for Mel MTP, CD44 and CD117 for Mel IL)^37^.

### Phototoxicity

**Figure 2:**
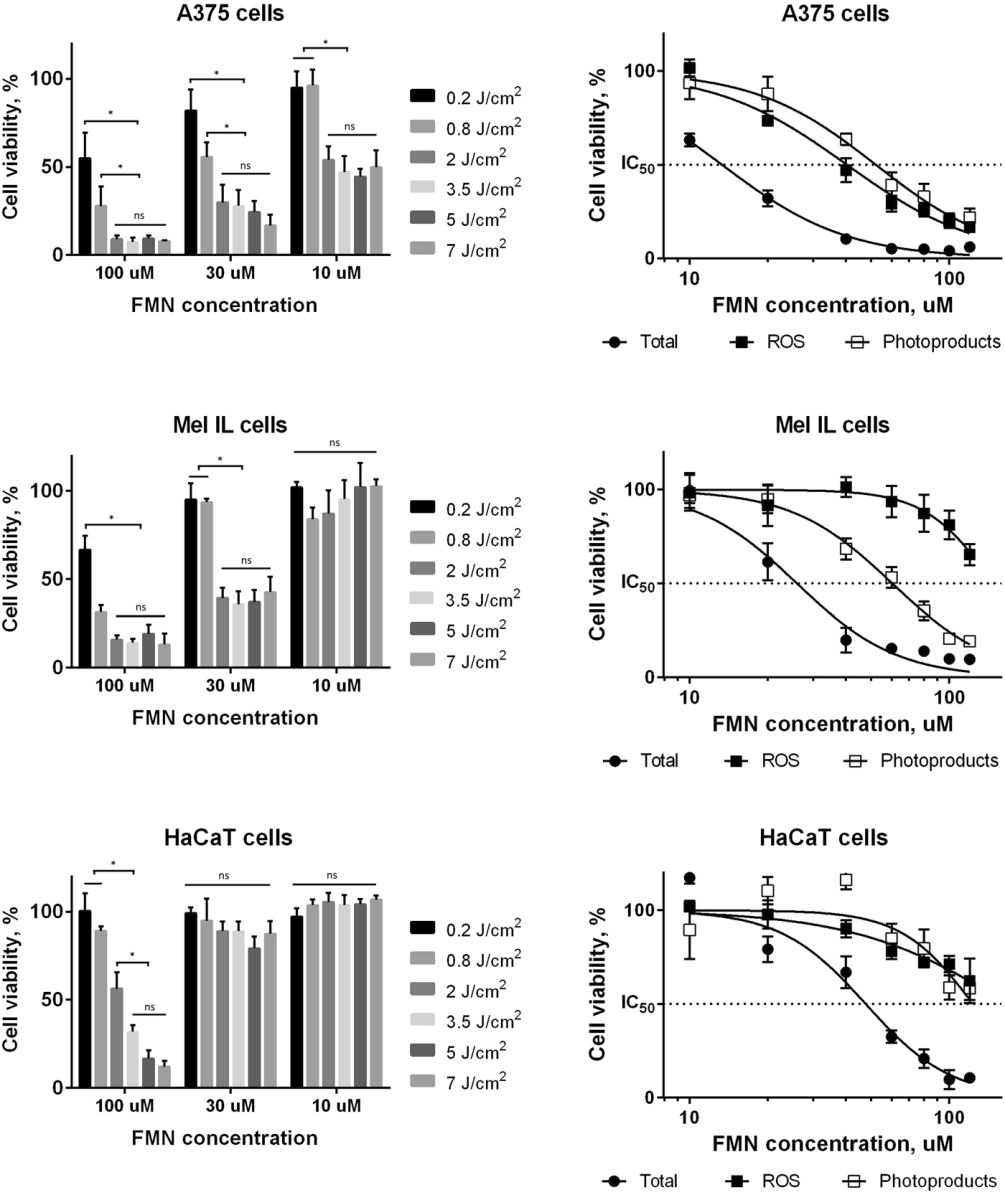
Viability of Mel IL, A375, and HaCaT cells after FMN photoactivation in exposure-dependent (left column) and FMN concentration-dependent at 5 J/cm^2^ (right column) manners, 48 h incubation after irradiation with 450 nm. ROS and FMN photoproducts toxicities were measured both separately (black and blank squares, respectively) and in total (black circles). Cells without any treatment were used as controls and taken as 100%. MTT assay, the data are the mean ± SD from at least three replicates. Statistical analysis was performed using non-parametric Mann-Whitney test, *p < 0.05.

The phototoxicity of FMN was studied using colorimetric MTT assay and fluorescent-based Annexin-FITC and PI staining. We tested dark toxicity of FMN at concentrations up to 5 mM, and no cell growth inhibition was found (Supplementary Data 2). It is important to note, that dark toxicity of commercially available PDT agents often achieves at micromolar concentrations, e.g. the IC_50_ values of photofrin (porfimer sodium) and temoporfin on human epidermoid carcinoma cells A431 are 5 μM and 8 μM, respectively^38,39^. On the other hand, the irradiation of cells incubated with FMN led to the cell death in concentration- and dose-dependent manner (Fig. 2). Thus, three cell lines of different FMN accumulation level, namely Mel IL (high FMN accumulation), A375 (medium FMN accumulation), and HaCaT (low FMN accumulation) were incubated with FMN and exposed to different dose of light centered at 450 nm. Clear irradiation dose-dependency was found for keratinocytes HaCaT at 100 µM of FMN, while 10 µM and 30 µM of FMN were lower toxic even at high irradiation dose. Contrary, for melanoma A375 cells we demonstrated irradiation dose-dependent curves even at 10 µM of FMN. It was found that light dose of 2 J/cm^2^ was enough to obtain maximum tumor cell growth inhibition, and higher light doses did not significantly increased the FMN cytotoxicity. This could be associated with the rapid FMN decomposition upon irradiation as it was described previously^40^. Our photoluminescence measurement showed about 70% FMN photobleaching at the laser dose 2 J/cm^2^. The IC_50_ values for tumor and normal cells after FMN photoactivation are summarized in Table 1. We can conclude that normal cells, in particular HaCaT keratinocytes, are more resistant to FMN-based PDT due to lower FMN accumulation. Since crosstalk between melanocytes and keratinocytes is important for melanoma progression^41^, the possibility of melanoma cells targeting and elimination without keratinocytes damage is of great interest. It is interesting that Mel IL cells were more resistant to FMN-based PDT than A375 cells despite higher FMN accumulation. This can be explained in terms of antioxidant activity and light absorption of melanin^42^ in highly pigmented Mel IL cells in comparison to amelanotic A375 cells^43^. Aiming to confirm this proposition, we quantified the melanin content in cells by measuring the absorption spectrum in DMSO cell lysate. Indeed, melanin concentration in Mel IL cells was 1.5–2 times higher compared to A375 cells (Supplementary Data 3). Melanin can protect melanoma cells from the ROS-mediated toxicity, but ROS is not the only toxicity factor. Thus, photoproducts of UV pre-irradiated riboflavin were demonstrated previously as modulators of B16F10 melanoma cells aggressiveness both *in vitro* and *in vivo* conditions^44^. Here, we evaluated the toxicity of ROS and the toxicity of photoproducts separately. For this, we pre-irradiated FMN solutions (450 nm, 5 J/cm^2^) and added these solutions to the cells next day. Since ROS have short life-time period, the toxicity of the pre-irradiated FMN solutions was mediated by FMN photoproducts only. To measure ROS-mediated toxicity, we replaced FMN-containing media with fresh RPMI-1640 immediately after irradiation (450 nm, 5 J/cm^2^). We demonstrated that ROS were more toxic for amelanotic A375 cells compared to the FMN photoproducts (IC_50_ values were 40.4 ± 4.0 µM and 52.2 ± 3.4 µM, respectively). Contrary, ROS were less effective against pigmented Mel IL cells than FMN photoproducts (IC_50_ values were more than 150 µM and 60.4 ± 7.1 µM, respectively). It is important that additive action of ROS and FMN photoproducts dramatically decreased FMN concentrations required for achieving 50% of cell viability.

**Table 1:** IC_50_ values for Mel Mtp, Mel IL, Mel Z, A375, and HaCaT cells after FMN photoactivation (450 nm, 5 J/cm^2^).

Cell line	IC_50_, μM		
	Total	ROS	FMN photoproducts
Mel MTP	24.2 ± 2.0	115.8 ± 11.8	103.4 ± 10.1
Mel IL	25.9 ± 1.3	>150	60.4 ± 7.1
Mel Z	29.0 ± 1.3	>150	>150
A375	13.3 ± 1.3	40.4 ± 4.0	52.2 ± 3.4
HaCaT	47.7 ± 4.6	>150	123.5 ± 10.2

Cells without any treatment were used as controls.

Photodynamic therapy can induce various cell death mechanisms, including apoptosis, necrosis, and autophagy, and these mechanisms can be concurrently occurred^45^. However, apoptosis is the preferred pathway for cancer cell elimination since it is a programmed cell death that does not induce unnecessary inflammation in tumor site. Here, we used Annexin-FITC and PI staining to determine apoptotic and necrotic cell populations after blue light irradiation of cells pre-incubated with FMN. Two melanoma cell lines, namely Mel MTP and Mel IL cells, were involved in this experiment, and a clear correlation between FMN concentration and cell death through apoptotic and necrotic pathway was found (Supplementary Data 4). Thus, apoptosis was specific for low (10 μM) and medium (30 μM) FMN concentration, while necrosis occurred mainly at high (100 μM) FMN dose.

### ROS measurement *in vitro*

**Figure 3:**
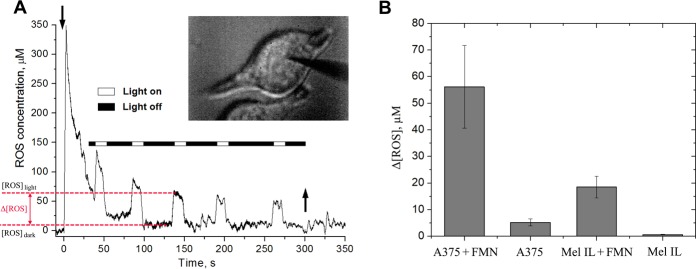
Electrochemical probe of ROS in melanoma cells: the measurement of ROS generated within A375 melanoma cells after pre-incubation with 100 µM FMN (**A**). Dark scale is for light off, white scale is for light on. The moments of electrode insertion into the cell and withdrawal from the cell are depicted with downward and upward arrows, respectively; the image of nanoelectrode inserted into the cell is in the upper right corner. The changes in ROS level under light irradiation were calculated according to the following formula: Δ[ROS] = [ROS]_light_ − [ROS]_dark_. The comparison of Δ[ROS] measured inside blue-light irradiated A375 and Mel IL melanoma cells, both FMN pre-incubated and control ones (**B**). Data are the mean ± SE, N = 5 for FMN-incubated cells and N = 3 for control cells.

As well known, ROS production is the most important factor for PDT efficacy^46^. At the same time, ROS-induced toxicity depends on cell-specific antioxidant defense system and tumor microenvironment. Therefore, direct measurement of ROS production in tumor cells in a real-time manner allows to predict the efficacy of the PDT. Earlier we reported a carbon disk-shaped nanoelectrode with platinum deposited on its tip as a tool for real time detection of ROS inside single cells with minimal cell disruption^47^. Recently, we increased nanoelectrodes stability and showed suitability for rapid routine screening of ROS-induced toxicity of magnetic nanoparticles^48^. Here, we were the first to implement a stable electrochemical probe based on platinized carbon nanoelectrodes for measuring intracellular ROS during PDT. For this purpose, A375 and Mel IL melanoma cells were incubated for 30 min with 100 μM FMN in darkness. After that, the unbound FMN was removed by triple washing with PBS to exclude the possible influence of extracellular FMN on the measurement accuracy. Then, the platinized nanoelectrode was carefully introduced by using the precise micromanipulator into the single cell under optical control. Nanoelectrode insertion led to an initial quick rise of ROS caused by mechanical stress. However, due to their nano-size (about 100 nm in diameter) and needle‐type shape, nanoelectrodes caused only minimal damage, and the ROS amount returned to the initial intracellular level within 10–50 s from the nanoelectrode incision. It should be noted, that in A375 cells the ROS burst was 10 times higher than in Mel IL cells (Supplementary Data 5A), which can be explained with the antioxidant activity of melanin in Mel IL cells. Light-induced ROS measurements started when ROS signal reached a plateau that can be discussed as a background ROS level typical for this cell line. Photoactivation was started by common fluorescent LED setup. It was found, that light irradiation led to a rapid growth of ROS production that could be quantified (Fig. 3A). Thus, A375 cells pre-incubated with 100 µM of FMN were able to produce 56 ± 15 µM of ROS, while Mel IL cells produced only 18.5 ± 4 µM of ROS (Fig. 3B). Contrary, switching off the light resulted in an immediately return to a background intracellular ROS level due to a short life-time period of ROS. This approach allowed us to monitor ROS production by electrochemical detection in a real-time manner before and during light irradiation which cannot be realized using conventional fluorescent-based probes. The irradiation of control cell (non-incubated with FMN) led to minor changes in ROS levels (5 ± 1 µM for A375 cells and 1 ± 0.5 µM for Mel IL cells), confirming FMN as a source of ROS. It should be noted, that ROS measurement with electrochemical probe is of high sensitivity and allows to detect ROS levels lower than 1 µM. Flavin derivatives are known to operate in photodynamic process through Type I (radical and radical anion species formation) and Type II (singlet oxygen formation) mechanisms. We believe that the predominant mechanism is Type II reaction, as well as in our previous work^31^, where we specifically detected singlet oxygen generated in the photosensitized FMN solution.

ROS production within melanoma cells after FMN irradiation was also measured with CellROX Deep Red fluorescent dye. Indeed, a clear correlation between FMN concentration and ROS production was confirmed for Mel IL and A375 cells (Supplementary Data 6). Thus, the fluorescent ROS signal for 10 µM of FMN was about 1.25-fold higher compared to control, while for 100 µM of FMN it was about 1.75-fold higher. Contrary to electrochemical detection in a real-time manner, fluorescent-based technique is suitable for reactive oxygen species determination in all treated cells during the irradiation cycle, so these two technics complemented each other.

### *In vivo* photodynamic therapy of melanoma

**Figure 4:**
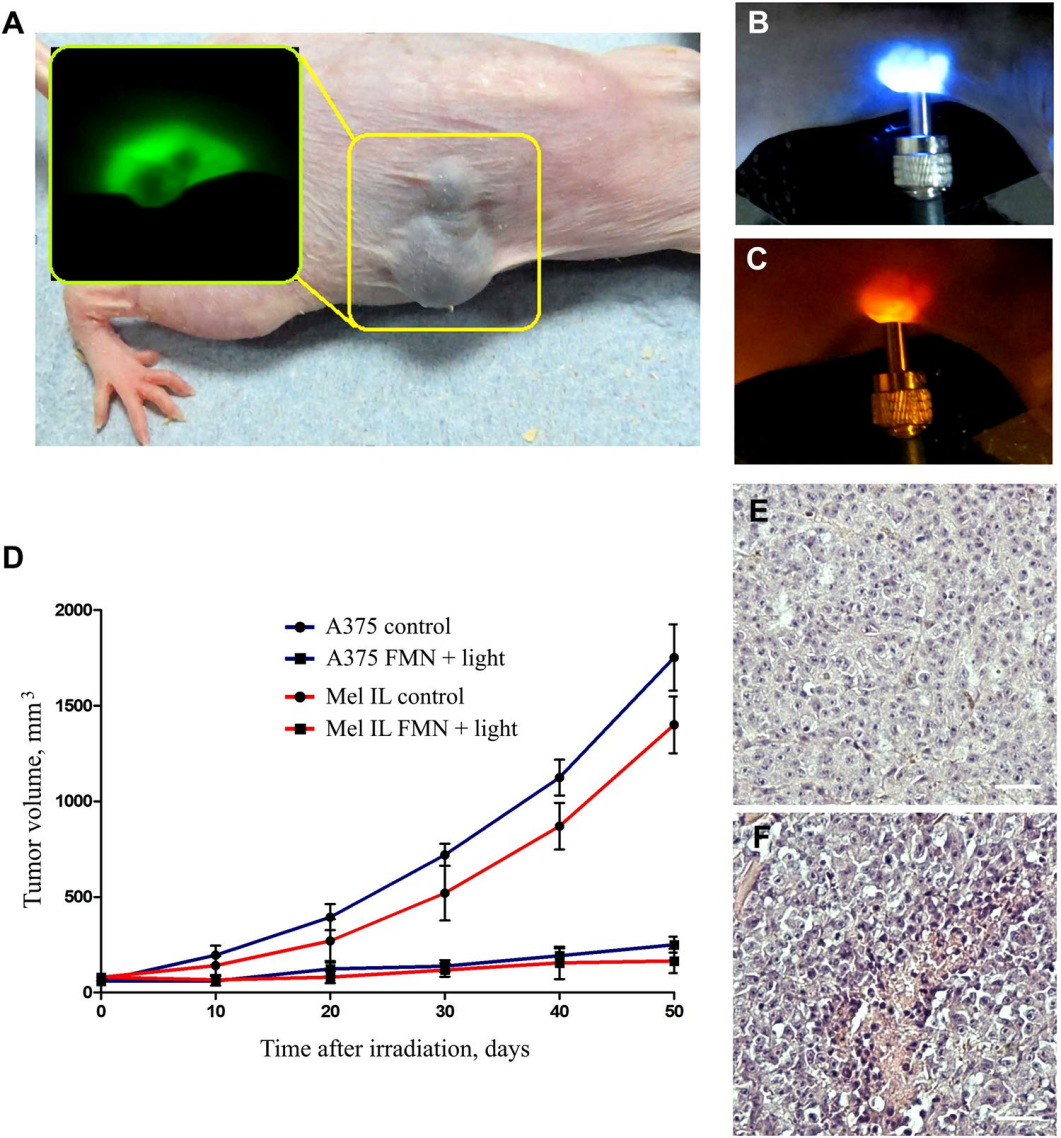
Photodynamic therapy of melanoma *in vivo*. The photo of mouse with high pigmented Mel IL melanoma xenograft (**A**), fluorescent image of the xenograft demonstrating FMN photoluminescence is in insert. The irradiation of low pigmented A375 xenograft site by blue-light (450 nm) coupled to the optical fiber (**B**). The photo of the same site taken through the red optical filter demonstrates FMN accumulation and photoactivation (red luminescence, 620–670 nm) in A375 xenograft (**C**). Tumor growth curves for Mel IL (red color) and A375 (blue color) xenografts during 50 days after PDT treatment (**D**), blank circles are for control group, black circles are for experimental group). Histology image analysis of Mel IL xenografts after PDT: control (**E**) and experimental (**F**) groups. Scale bar is 100 µm.

We performed a series of experiments on mice bearing melanoma xenograft aiming to demonstrate the *in vivo* efficacy of FMN. Two cell lines, namely A375 and Mel IL, were chosen for research. Melanoma cells suspension in Matrigel (2 × 10^6^ cells per injection) was implanted subcutaneously into the right flank of the mice. The experimental treatment started when melanoma xenografts reached 100–120 mm^3^. The animals were administrated intravenously with 0.1 mg/kg FMN and incubated for 1 h, followed by irradiation with a blue light at 450 nm of the dose 20 J/cm^2^ for 15 min. Animals, which were administered with FMN without 450 nm light exposure, were used as the control. The *in vivo* FMN-induced PDT efficacy was assessed by measuring the tumor sizes over a period of 50 days, with the results presented in Fig. 4. Tumor growth curves showed a progression for the control group, whereas no significant growth was observed for PDT treatment groups. The tumor growth inhibition was estimated as 85% of volume for A375 and 89% of volume for Mel IL on the day 50 after PDT treatment. Histology analysis of the paraffin-embedded tumor sections 24 h after PDT demonstrated intense functional disturbances with vasodilation and red blood cell extravasation. In both A375 and Mel IL specimens most of the cells in the tumor nodules appeared to be damaged (Fig. 4, Supplementary Data 7).

Since metastatic formation is a frequent complication of melanoma, we performed experiments to evaluate the influence of FMN-based PDT therapy on distant tumor site. We tested the impact of FMN associated PDT treatment on model of distant tumors^49^, when only one of two subcutaneous tumors was irradiated. B16 F10 cells were inoculated subcutaneously into C57BL/6 mice (see Experimental for details) and grown until tumors reached 60 ± 16 mm^3^ in volume both the left and right flanks. In the PDT treated groups, after intravenous injection of FMN (150 μl, 10 mg/ml) and incubation for 1 h only left-flank tumors were irradiated with a blue light (dose 20 J/cm^2^). Laboratory animals treated by the laser light without FMN injection and FMN injection without laser light treatment were used as a control groups. We observed the growth of both treated and distant tumors within 16 days (Supplementary Data, Fig. 8) and found that untreated right-flank tumors in the PDT groups grew slower than in the control. Decrease in distant tumor growth rate comparing to the control tumors was estimated as 20% of inhibition for single PDT procedure and 30% for double PDT procedure. We assume that tumor PDT treatment has immunological impact on growth rate of the other one. Although the observed effect requires further study, we speculate that FMN-based PDT therapy can be promising treatment to prevent metastasis formation and reduce the risk of recurrence with combining standard surgical resection.

## Conclusions

We demonstrated that a non-toxic flavin mononucleotide manifests as an effective photosensitizer for melanoma treatment. A selective accumulation of FMN in melanoma cells was shown. Evaluation of the FMN phototoxicity as a result of additive action of ROS and FMN photoproducts revealed that the IC_50_ values were in a range of 10–30 µM for melanoma cells. An electrochemical probe for real-time single cell monitoring of ROS production was firstly proposed to assess the efficacy of FMN photoactivation *in vitro*. Melanoma xenograft regression in mice (85–90% of volume within 50 days) was observed as a result of systemic intravenous injection of FMN followed by 450 nm blue-light photoactivation. In summary, all of these data represent the broad prospects for FMN-based therapy as a promising approach for clinical applications in a nearest future.

## Materials and Methods

### Materials

Flavin mononucleotide (riboflavin-5′-phosphate) was obtained from Pharmstandard (Russia). Phosphate buffered saline (PBS, pH 7.4) was purchased from Lonza (Switzerland). RPMI-1640 cell growth medium, L-glutamine, and Penicillin-Streptomycin (Pen Strep, 100x) were purchased from Gibco (UK), fetal bovine serum (FBS) was from HyClone (USA). MTT dye (3-(4,5-dimethylthiazol-2-yl)-2,5-diphenyltetrazolium bromide), dimethyl sulfoxide (DMSO), Versene solution, and paraformaldehyde were from Sigma (USA). CellROX Deep Red fluorescent dye, Annexin V-FITC Apoptosis Detection Kit, Hematoxylin and Eosin were from Thermo Fisher Scientific (USA). BD Matrigel™ Basement Membrane Matrix was from BD Biosciences (USA).

### Cell culture

Human melanoma Mel Z, Mel IL, and Mel MTP cells were obtained from the tumor material of patients undergoing treatment at the N.N. Blokhin National Medical Research Center of oncology^50^. BJ-5ta fibroblasts and breast cancer SK-BR-3 cells were from ATCC. The other cells were kindly provided by Drs. E. Svirchevskaya and E. Kovalenko (Shemyakin - Ovchinnikov Institute of Bioorganic Chemistry RAS, Moscow, Russia). All cells were grown in RPMI-1640 growth medium supplemented with 10% FBS, 2 μМ L-glutamine, 100 μg/mL streptomycin, and 100 U/mL penicillin at 37 °C in a 5% CO_2_ humidified atmosphere. The medium was replaced every 3–4 days. Melanin content was estimated by measuring the absorption spectrum in DMSO cell lysates (10^6^ cells per ml) using a Cary 50 UV-Vis spectrophotometer.

### Flow cytometry

Cells were harvested with Versene solution, pelleted by centrifugation at 300 g for 5 min, and resuspended in RPMI-1640 media without serum to the final concentration of 10^6^ cells per ml. Then, 100 μl of the cell suspension were transferred to the light-protected tubes for flow cytometry. Appropriate working solutions of FMN in RPMI-1640 medium were prepared immediately prior to the experiments. These FMN solutions were added to the tubes, and the final FMN concentration was 10 µM, 30 µM, and 100 µM. Then, the cells were incubated in the dark in a CO_2_ incubator for 30 minutes. At the end of the incubation, 1 ml of cold PBS was added to each tube, and the cells were centrifuged (300 g, 4 °C) for 5 min. The supernatant was removed and the cells were resuspended in 300 μl of cold PBS and transferred to ice. Fluorescence was examined using a flow cytometer NovoCyte 2000R (ACEA Biosciences) and NovoExpress v.1.2.4 software. Fluorescence was measured on a channel corresponding to FITC fluorescence, and at least 10,000 events were examined for each sample. To estimate the accumulation level of FMN in the cells, the median fluorescence values in each sample were determined, and then the relative fluorescence level (F) was calculated according to the following formula:

$${\rm{F}}={{\rm{FLUO}}}_{{\rm{sample}}}-{{\rm{FLUO}}}_{{\rm{background}}}$$

### Confocal microscopy

Cells were seeded in a 96-well plates (5 × 10^3^ cells per well) followed by overnight incubation. Then, 100 μL of appropriate working solutions of FMN (10 µM, 30 µM, and 100 µM) in RPMI-1640 medium were added to cells and the plate was incubated in the dark in a CO_2_ incubator for 30 minutes. After that, cells were washed 3 times with cold PBS and additionally stained with Hoechst 33258 dye (50 μM) for 15 min. Optical images and fluorescence intensity data were acquired using InCell Analyzer 6000 and In Cell Analyzer Workstation software v.3.7.3 (GE Healthcare, USA).

### Cytotoxicity studies

Cells were seeded in a 96-well plate (5 × 10^3^ cells per well) followed by overnight incubation. Appropriate working solutions of FMN in full RPMI-1640 medium were prepared immediately prior to the experiments. Then, the medium was removed from the 96-well plate and FMN solution aliquots (100 µl) were added in each well. To induce FMN photoactivation, the plates were treated with light at 450 nm, irradiation dose 0.2 J/cm^2^–7 J/cm^2^. Cells without any treatment were used as controls (100%). Cell viability was determined by MTT assay after 48 h incubation. For this purpose, cells were treated with MTT solution (0.5 mg/ml in RPMI-1640) for 3 h. Then the medium was replaced with 100 µl of DMSO in order to dissolve formed formazan crystals. The optical density (OD) at 570 nm was measured using Varioskan Flash reader (Thermo Scientific, USA). The viability of cells (V) after FMN treatment was expressed in % compared to the control according to the following equation:

$${\rm{V}}=({{\rm{OD}}}_{{\rm{sample}}}-{{\rm{OD}}}_{{\rm{background}}})/({{\rm{OD}}}_{{\rm{control}}}-{{\rm{OD}}}_{{\rm{background}}})\times 100 \% .$$

Aiming to determine the cytotoxicity of FMN photoproducts, appropriate working solutions of FMN in full RPMI-1640 medium were irradiated at 450 nm (5 J/cm^2^) and incubated overnight. Then these FMN aliquots (100 µl) were added to the cells and incubated for 48 h. MTT assay was performed as described above.

Aiming to determine ROS-mediated phototoxicity, FMN solution aliquots (100 µl) were added in each well and treated with light at 450 nm (5 J/cm^2^). FMN-containing media was removed immediately after the treatment and cells were incubated in fresh RPMI-1640 media for 48 h. MTT assay was performed as described above.

### Apoptosis and necrosis assay

Cells were seeded in a 96-well plates (5 × 10^3^ cells per well) followed by overnight incubation. Then, 100 μL of appropriate working solutions of FMN (10 µM, 30 µM, and 100 µM) in RPMI-1640 medium were added to cells and the plate was incubated in the dark in a CO_2_ incubator for 30 minutes. After that, cells were irradiated using 450 nm laser at a dose of 5 J/cm^2^. Then, the cells were consistently washed with cold PBS and binding buffer and stained with Annexin V-FITC, propidium iodide (PI), and Hoechst 33258 at room temperature in binding buffer for 15 min. Optical images and fluorescence intensity data were acquired using InCell Analyzer 6000 and In Cell Analyzer Workstation software v.3.7.3 (GE Healthcare, USA).

### Electrode production

The carbon nanoelectrodes were etched in a 0.1 M NaOH, 10 mM KCl solution during 30–40 cycles for 10 seconds each to create a cavity in the carbon surface. The applied potential was V-shaped with amplitude of 1.5–2 V. Platinum deposition was carried out by sweeping the potential from 0 to −1500 mV vs Ag/AgCl in a solution containing 2 mM chloroplatinic acid (H_2_PtCl_6_, Sigma). The cyclic voltammograms in 1 mM ferrocene methanol (FcMeOH, Sigma) were obtained for the initial carbon electrode, for the electrode with nanocavities and with platinum to control the production process. The deposition of Pt only slightly increased the effective geometric surface area of the nanoelectrode (as evidenced by the voltammogram for the oxidation of 1 mM FcMeOH), but dramatically enhanced its catalytic activity toward ROS reduction. The nanoelectrode was polarized at 600 mV vs Ag/AgCl (for the diffusion-limited detection of H_2_O_2_) and manually approached to the liquid or the surface of the single cancer cell. For the calibration we used solutions with different concentration of H_2_O_2_ (Sigma) in range of 10^−7^–10^−4^ M.

### ROS measurement with electrochemical probe

The results were obtained as it was described earlier^47^ with some alterations. Thus, the introduced modification of electrodes used in this work is the better adhesion of platinum to carbon electrode due to etching a cavity in carbon layer before Pt deposition. Commercially available disk, shaped carbon nanoelectrodes isolated in quartz (ICAPPIC Limited, UK) with diameter 100–500 nm, were used for production of sensors for ROS detection. The relatively inert carbon surface of the electrode was further functionalized for the detection of redox-active species. An electrodeposited platinum layer enhances the electrocatalytic activity by drastically reducing the overpotential produced by the reduction of reactive oxygen species. To enhance the adhesion of the platinum to the carbon plug, we etched a nanocavity in the carbon electrode. We have used an electrochemical method to create tiny notches in the tips of carbon nanoelectrodes^51^. Fabrication of platinum nanosensors was performed in two stages: etching in alkaline solution and platinization. All stages were precisely controlled by electrochemical measurements. We believe that the nanoelectrode is sensitive both to short-lived and long-lived ROS forms. Recently we demonstrated that the singlet oxygen production in aqueous solution of flavin mononucleotide (FMN) is induced by blue light irradiation. Based on this result we demonstrate (Supplementary data 9) response of carbon nanoelectrode polarized at +800 mV vs Ag/AgCl on ROS production in aqueous solution of FMN under blue light irradiation.

Cells were seeded in a Petri dish followed by overnight incubation. Then, the cells were incubated for 30 min with 100 μM FMN in darkness and washed 3 times with PBS. Then, the platinized nanoelectrode was carefully introduced by using the precise micromanipulator into the single cell under optical control with inverted microscope. For electrode manipulation and adjustment we used micromanipulator PatchStar (Scientifica, UK). We collected a cyclic voltammetric data with patch-clamp amplifier Model 2400 (A-M Systems, USA). Recording of the signal was performed with multifunctional I/O device USB-6211 (National instruments, USA) and computer program WinWCP. All measurements were accomplished on the table of inverted microscope Nikon (Japan). In all measurements Ag/AgCl electrode was used as a reference electrode.

### ROS measurement with fluorescent probe

Evaluation of the cytoplasmic ROS was performed using CellROX Deep Red fluorescent probe (Molecular Probes/Thermo). Briefly, Mel IL and M-3 melanoma cells (5 × 10^3^) were seeded in 96-well plates (Nunc, Denmark) and incubated with 2.5 µM of CellROX Deep Red in full RPMI-1640 media for 30 min and washed in PBS three times. Then 100 μL of appropriate working solutions (10 µM, 30 µM, and 100 µM) of FMN in RPMI-1640 medium without Phenol Red were added to cells and plate was incubated in the dark in a CO_2_ incubator for 30 minutes. After that, cells were irradiated using 450 nm laser at a dose of 5 J/cm^2^. Cell nuclei were additionally stained by 50 μM of Hoechst 33258 dye solution for 15 min. Optical images and CellROX Deep Red fluorescence intensity data of Mel IL and A375 melanoma cells were acquired using InCell Analyzer 6000 and In Cell Analyzer Workstation software v.3.7.3 (GE Healthcare, USA).

### Melanin content determination

We use routine assay for spectrophotometry melanin determination with minor changes^52^. Four melanoma cell lines: Mel MTP, Mel IL, Mel Z, A375 and two normal (human keratinocytes HaCaT and human skin fibroblasts BJ-5ta) cell lines were counted and pelleted in order to have a final concentration of 2 × 10^6^ cells/ml. For intracellular melanin content determination, cell pellets were dissolved in 1 M NaOH containing 10% (v/v) DMSO and incubated at 80 °C for 1 h. After incubation the lysates were centrifuged (3000 g for 5 min) and the absorbance was measured in cell-free media at 405 nm. All the results are expressed as mean value of absorbance intensities normalized to the melanin content in fibroblasts ± standard deviation (SD) of three independent experiments. Statistical analysis was performed with GraphPad Prism version 5.0 (GraphPad Software, La Jolla California USA).

### Animal experiments

#### Therapy of primary tumors

Male Balb/c nu/nu mice aged 6–7 weeks were purchased from animal farm of Shemyakin–Ovchinnikov Institute of Bioorganic Chemistry, Russian Academy of Sciences, and housed under controlled environmental conditions in vented animal cabinet A-Box 80 (Noroit, France) at a 12 h dark-light cycle and allowed access to sterile water and SPF-mouse chow freely. All animal experiments were performed in accordance with European and Russian national guidelines for animal experimentation and were approved by the animal and ethics review committee of the FSBSI “N.N. Blokhin NMRCO”, reference number 2017-034.

To establish a xenograft mouse model, Mel IL and A375 cells were harvested with Versene solution, pelleted by centrifugation at 300 g for 5 min, and resuspended in RPMI-1640 media without serum. Melanoma cell suspension was mixed (1:1 volume) with Matrigel (BD Biosciences), and the obtained cell suspension (2 × 10^6^ cells per injection) was implanted subcutaneously into the right flank of the mice to ensure successful tumor initiation and tumor growth measurements.

Administration of FMN started on day 10 after inoculation when tumor size reached 100–120 mm^3^. FMN solution (150 μl, 10 μg/ml) was injected into the mice intravenously through a retro-orbital sinus, and the tumors were irradiated with blue-light at 450 nm for 15 min to the final dose of 20 J/cm^2^.

Tumor volume was estimated by the standard method of calipation and was calculated by the following formula, assuming a hemi-ellipsoid shape:

$${{\rm{V}}}_{{\rm{tumor}}}={\rm{length}}\times {({\rm{width}})}^{2}/2$$

Antitumor activity of photoactivated FMN was determined by evaluating the tumor growth inhibition rate (TGI%) calculated as:

$${\rm{TGI}} \% =({{\rm{TG}}}_{{\rm{control}}}-{{\rm{TG}}}_{{\rm{test}}})/{{\rm{TG}}}_{{\rm{control}}}\times \mathrm{100} \% $$

#### Photodynamic therapy of distant tumors

We used C57BL/6 mice at 6–7 weeks of age, purchased from animal farm of Shemyakin–Ovchinnikov Institute of Bioorganic Chemistry, Russian Academy of Sciences. All animal experiments were performed in accordance with European and Russian national guidelines for animal experimentation and were approved by the animal and ethics review committee of the FSBI “N.N. Blokhin NMRCO”, reference number 2017-034.

B16-F10 mouse melanoma cells were harvested with Versene solution, pelleted by centrifugation at 300 g for 5 min, and resuspended in RPMI-1640 media without serum. Melanoma cell suspension was mixed (1:1 volume) with Matrigel (BD Biosciences), and the obtained cell suspension (5 × 10^5^ cells per injection) was implanted subcutaneously into the right and left flank of the mice to ensure successful tumor initiation and tumor growth measurements.

Administration of FMN started on day 4 after inoculation when tumor size reached 60 ± 16 mm^3^ FMN solution (150 μl, 10 mg/ml) was injected into the mice intravenously through a retro-orbital sinus, and the tumors were irradiated with blue-light at 450 nm for 15 min to the final dose of 20 J/cm^2^. Two control groups were used: FMN injection with no light irradiation; laser irradiation with no FMN injection. Tumor volume and antitumor activity of photoactivated FMN was estimated as it was described above.

### Hematoxylin and Eosin staining

Animals were sacrificed by cervical dislocation in 24 hours after PDT. All harvested tumors were fixed with 4% paraformaldehyde for 24 h and then embedded with paraffin. Tissue sections (4 µm thick) were stained with hematoxylin and eosin.

### Statistical analysis

All experiments were performed in triplicate unless otherwise noted, and each experiment was repeated three times independently. Data are expressed as mean ± SD. Differences were considered to be statistically significant when P values were less than 0.05. All statistical analyses were performed with GraphPad Prism software (GraphPad Software, La Jolla, San Diego, CA, USA).

## Supplementary information

Supplementary information

## Data Availability

No datasets were generated or analyzed during the current study.
