# Supplementary material for: Photodynamic therapy of melanoma by blue-light photoactivation of flavin mononucleotide
Source: Sci Rep. 2019 Jul 4;9:9679. doi: 10.1038/s41598-019-46115-w (PMC6609768; doi:10.1038/s41598-019-46115-w)
Supplement: Supplementary file 1 — Supplementary information [file 41598_2019_46115_MOESM1_ESM.pdf]

Supplementary information

Photodynamic therapy of melanoma by blue-light photoactivation of flavin mononucleotide

Akasov R.A.[1,2,3,7]*, Sholina N.V.[1,3,4], Khochenkov D.A.[3,4,8], Alova A.V.[5], Gorelkin P.V.[6], Erofeev A.S.[5,7], Generalova A.N.[2,3], and Khaydukov E.V. [3,1,9]

[1] I.M. Sechenov First Moscow State Medical University, 119991, Trubetskaya str. 8-2, Moscow, Russia

[2] Shemyakin - Ovchinnikov Institute of Bioorganic Chemistry Russian Academy of Sciences, 117997, Miklukho-Maklaya str. 16/10, Moscow, Russia

[3] Federal Scientific Research Center «Crystallography and Photonics» Russian Academy of Sciences, 119333, Leninskiy Prospekt 59, Moscow, Russia

[4] FSBSI "N.N. Blokhin National medical research center for oncology" of Ministry of Health of the Russian Federation, 115478, Kashirskoe Shosse 24,Moscow, Russia

[5] Lomonosov Moscow State University, 119991, Leninskiye Gory 1-3, Moscow, Russia

[6] Medical Nanotechnology LLC, Stroiteley 4-5-47, 119311 Moscow, Russia

[7] National University of Science and Technology «MISIS», Leninskiy Prospect 4, 119991 Moscow, Russia

[8] Togliatti State University, 445020, Belorusskaya str. 14, Togliatti, Russia

[9] Volgograd State University, 400062, Universitetskiy Prospect, 100, Volgograd, Russia

* roman.akasov@gmail.com

Supplementary data

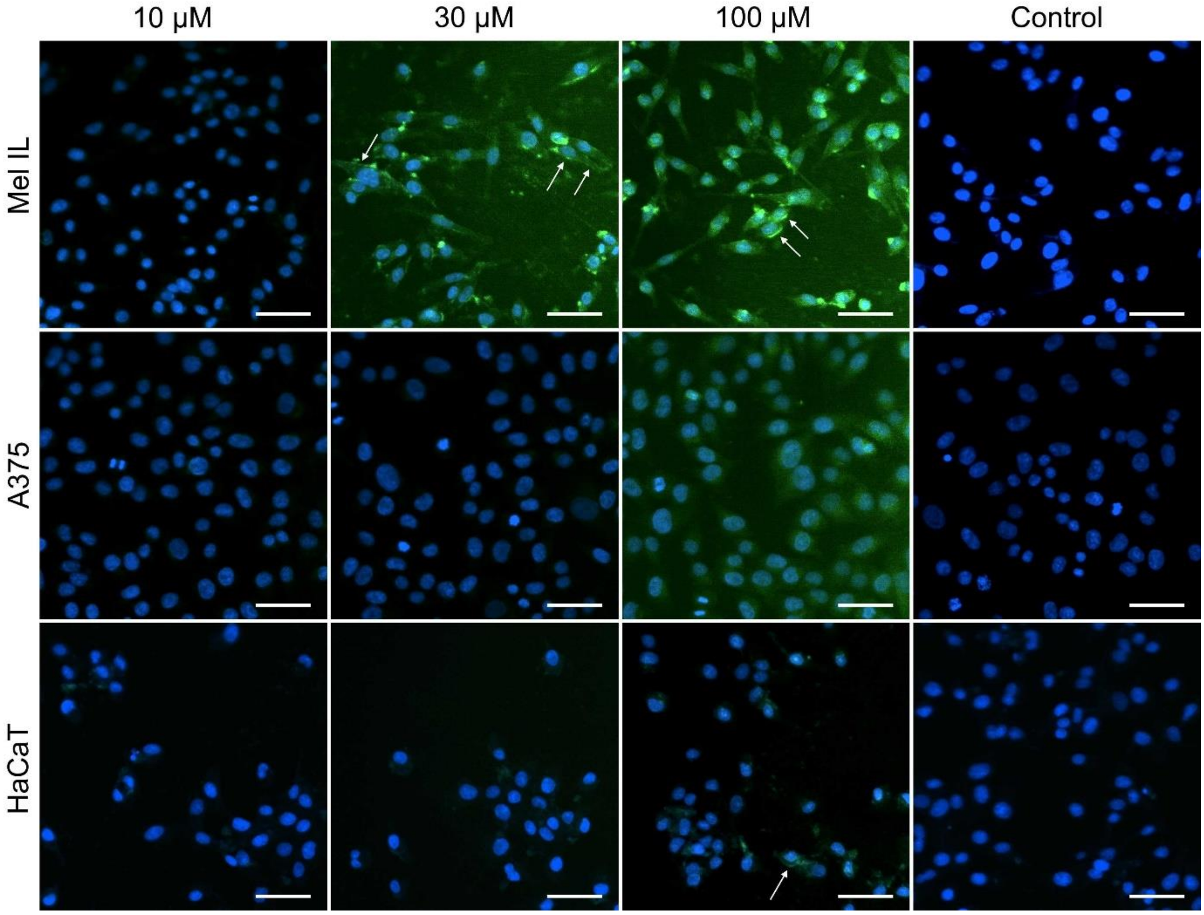

Supplementary data 1.FMN accumulation in melanoma (Mel IL and A375) and normal (HaCaT keratinocytes) cells: confocal microscopy images, 30 min of incubation. Cell nucleuses are in blue (Hoechst 33258), FMN is in green. White arrows show the FMN accumulation on the cell membrane. Scale bar is 50 μm.

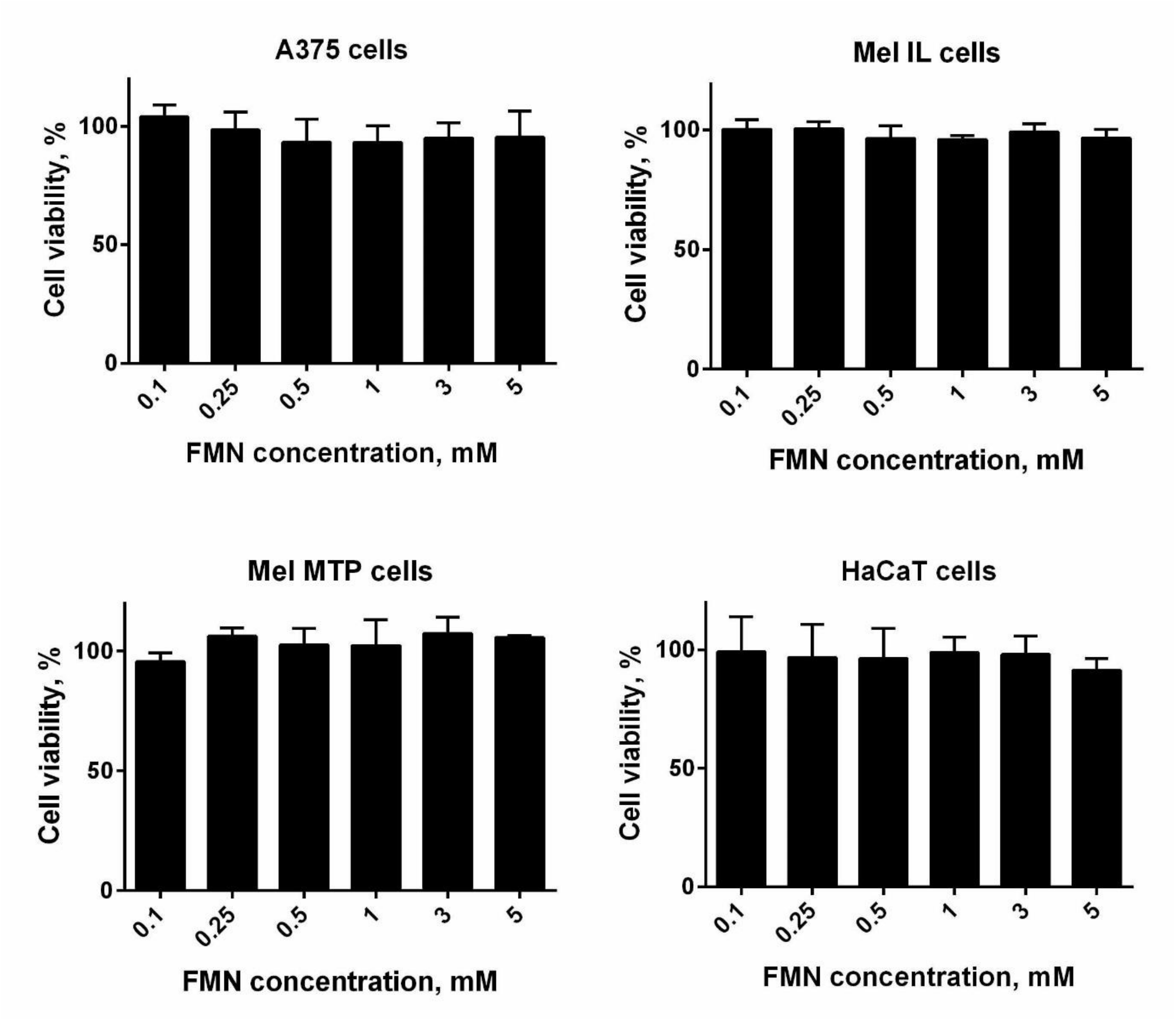

Supplementary data 2. Viability of A375, Mel IL, Mel MTP, and HaCaT cells after 24 h incubation with FMN in dark. Cells without any treatment were used as controls and taken as 100%. MTT assay, the data are the mean ± SD from at least three replicates.

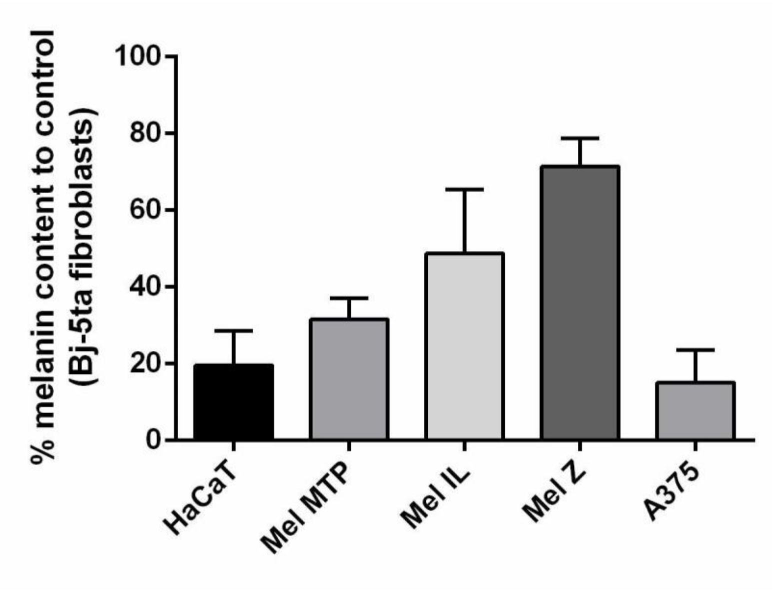

Supplementary data 3. Melanin content in the studied cell lines. The results are expressed as mean value of absorbance intensities normalized to the melanin content in fibroblasts ± standard deviation (SD) of three independent experiments.

Supplementary data 4. Percentage of apoptotic and necrotic cells after FMN photoactivation (30 min incubation with FMN followed by 5 J/cm^2 irradiation dose) in Mel MTP and Mel IL cells. Annexin V-FITC (An) and propidium iodide (PI) staining, the data are the mean ± SD.

%	Mel MTP				Mel IL			
	An-PI-	An+PI-	An-PI+	An+PI+	An-PI-	An+PI-	An-PI+	An+PI+
100 μM FMN + light	4.6±3.2	46.1±4.6	2.6±1.2	46.5±6.5	5.2±1.9	47.4±3.1	4.0±1.5	43.4±5.4
30 μM FMN [illegible]	15.7±2.3	66.7±5.0	1.5±0.2	16.1±5.8	32.3±1.8	46.2±6.3	1.8±0.2	19.8±5.2
10 μM FMN [illegible]	11.6±2.4	74.1±0.6	1.3±0.2	12.9±2.6	25.3±3.4	61.5±3.7	1.5±1.1	11.7±1.7
no FMN [illegible]	53.3±2.1	46.6±2.0	0.1±0.1	0.1±0.1	53.0±4.7	43.9±2.7	1.0±0.8	2.1±1.8
no FMN, [illegible]	87.5±2.5	12.3±2.6	0.2±0.1	0.1±0.1	82.2±3.7	16.5±3.9	1.1±0.3	0.2±0.2

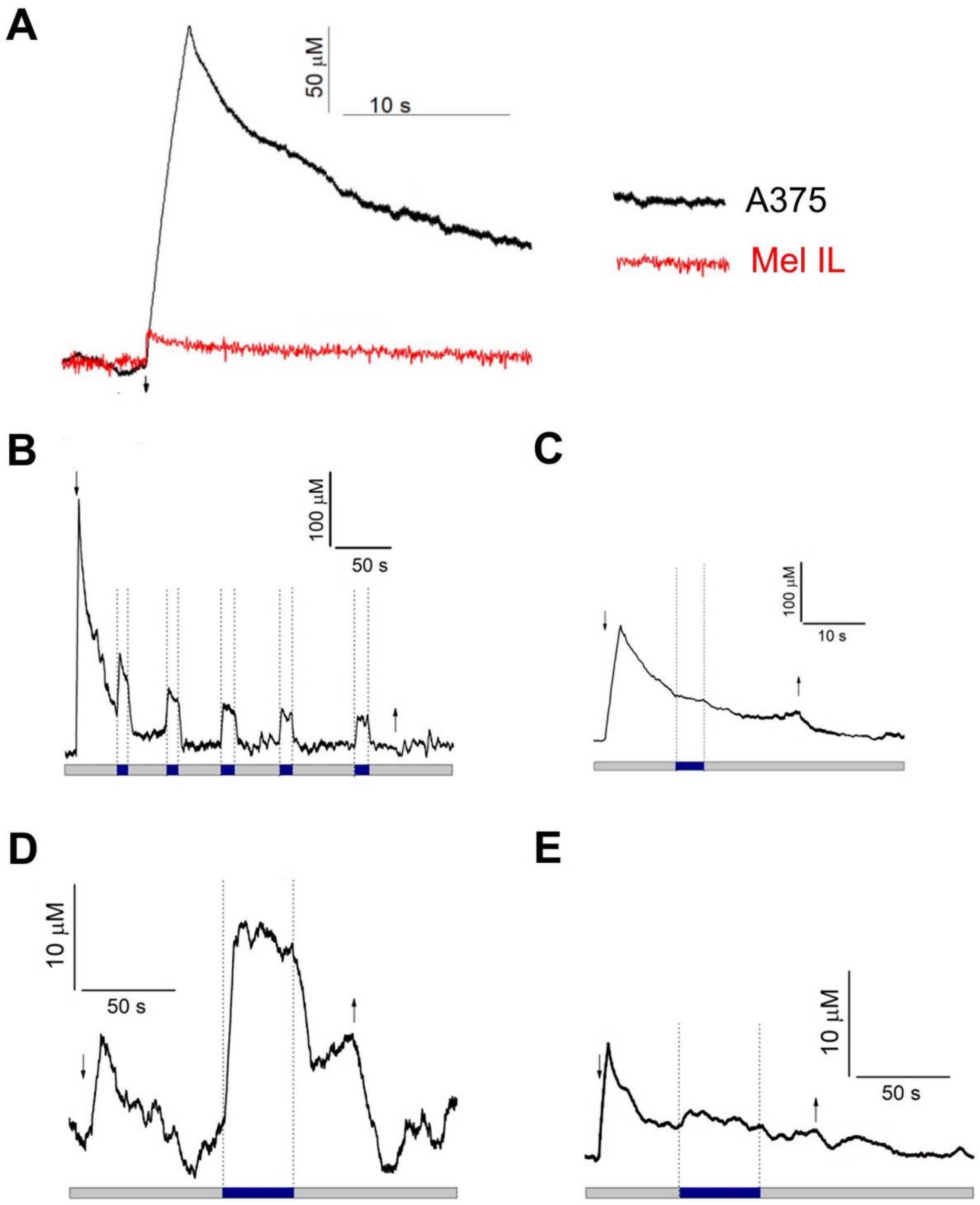

Supplementary data 5. ROS production in Mel IL and A375 cells measured using electrochemical probe: mechanical stress-induced ROS burst after the insertion of platinized carbon nanoelectrode into a cell (A). ROS kinetics in A375 cells pre-incubated with 100 μM FMN (B) and without FMN pre-incubation (C). ROS kinetics in Mel IL cells pre-incubated with 100 μM FMN (D) and without FMN pre-incubation (E). Grey scale is for light off, while blue scale is for light on. Downward and upward arrows mark the insertion and the removal of the nanoelectrode from the cell. The periods of light irradiation are shown with blue boxes and dashed lines.

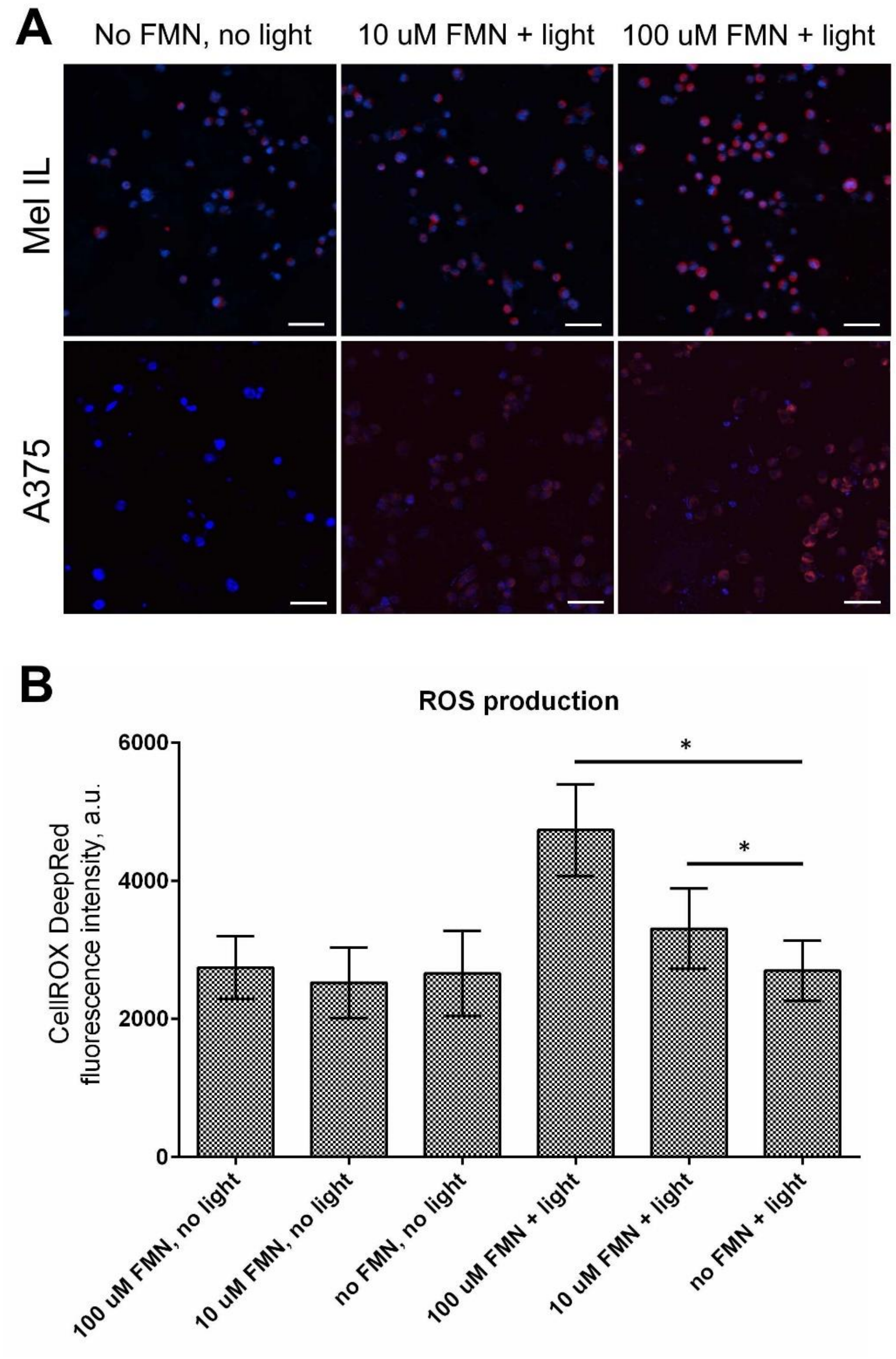

Supplementary data 6. ROS production in Mel IL and A375 cells measured using semi-quantitative CellROX fluorescent assay: confocal microscopy images (A) and image-based ROS level measurement in Mel IL cells (B). Cell nucleuses are in blue (Hoechst 33258), CellROX is in red. Cells were exposed to 450 nm light, 5 J/cm^2. Scale bar is 50 μm. The data are the mean ± SD. Statistical analysis was performed using non-parametric Mann-Whitney test, * $p < 0.05$.

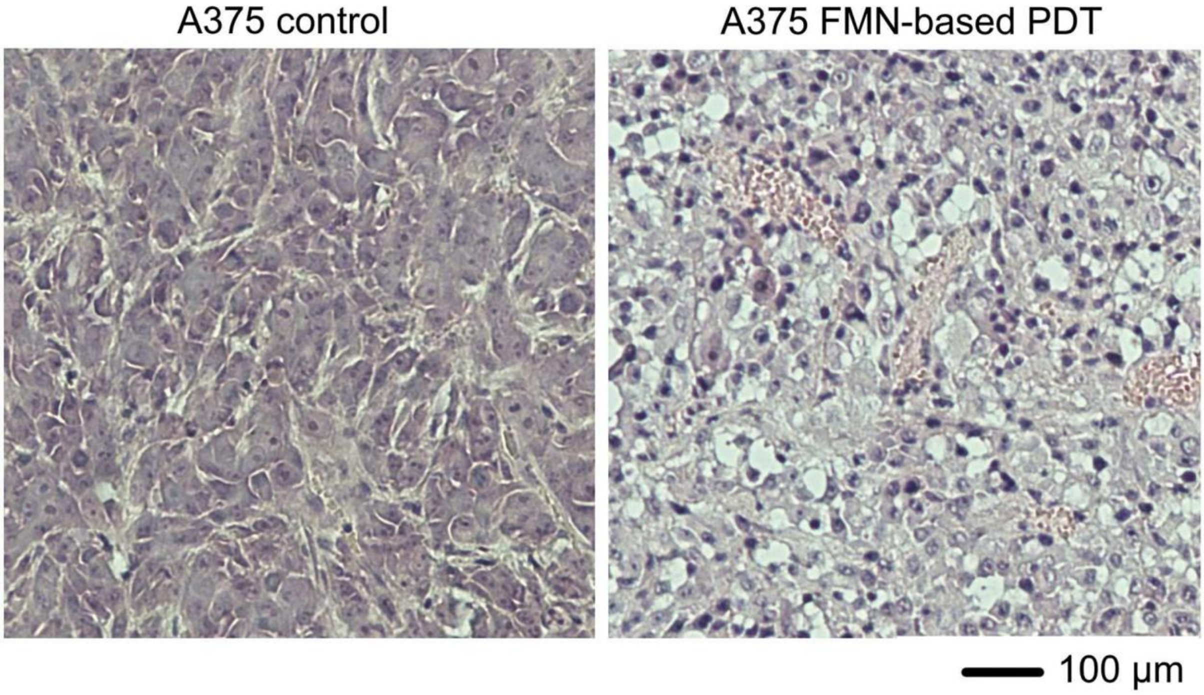

Supplementary data 7. Histology image analysis of A375xenografts after PDT: control (left panel) and experimental (right panel) group.

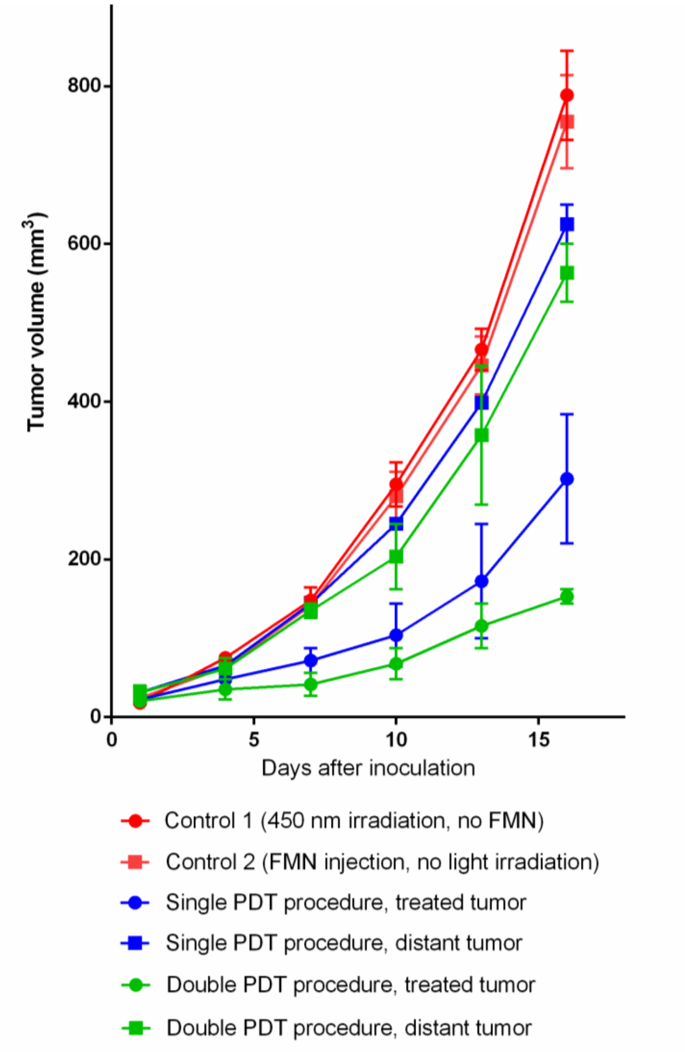

Supplementary data 8. FMN-based in vivo photodynamic therapy of B16 F10 melanoma. Tumor growth curves for controls (red color), single PDT procedure (blue color), and double PDT procedure (green color). Circles are for treated tumors, squares are for distant tumors.

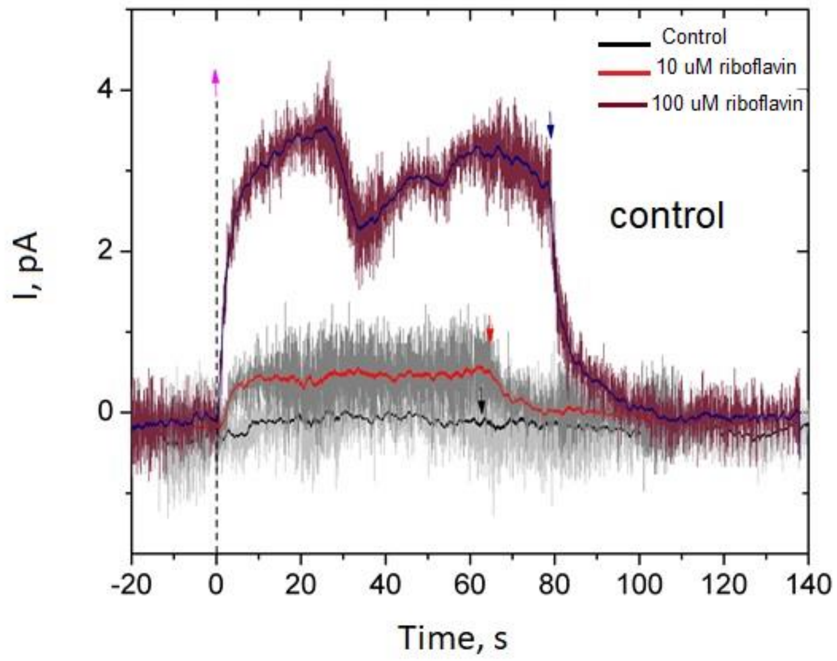

Supplementary data 9. Response of a nanoelectrode polarized at +800 mV vs Ag/AgCl on ROS production with FMN under blue light irradiation
